# Multiprotein Complexes of Plant Glycosyltransferases Involved in Their Function and Trafficking

**DOI:** 10.3390/plants14030350

**Published:** 2025-01-24

**Authors:** Ning Zhang, Jordan D. Julian, Olga A. Zabotina

**Affiliations:** Roy J. Carver Department of Biochemistry, Biophysics and Molecular Biology, Iowa State University, Ames, IA 50011, USA; ningz@iastate.edu (N.Z.); jjulian@iastate.edu (J.D.J.)

**Keywords:** glycosyltransferases, ER-Golgi trafficking, homodimer and heterodimer, assembly of the protein complexes, protein structure

## Abstract

Plant cells utilize protein oligomerization for their functions in numerous important cellular processes. Protein-protein interactions are necessary to stabilize, optimize, and activate enzymes, as well as localize proteins to specific organelles and membranes. Glycosyltransferases—enzymes that attach sugars to polysaccharides, proteins, lipids, and RNA—across multiple plant biosynthetic processes have been demonstrated to interact with one another. The mechanisms behind these interactions are still unknown, but recent research has highlighted extensive examples of protein-protein interactions, specifically in the plant cell wall hemicellulose and pectin biosynthesis that takes place in the Golgi apparatus. In this review, we will discuss what is known so far about the interactions among Golgi-localized glycosyltransferases that are important for their functioning, trafficking, as well as structural aspects.

## 1. Introduction

Glycosyltransferases (GTs) represent a diverse and crucial family of enzymes in plants, playing a fundamental role in the biosynthesis and modification of various plant compounds. GTs catalyze the transfer of monomeric sugars from activated sugar donor molecules to specific acceptor substrates, forming glycosidic bonds. They are integral to the biosynthesis of complex carbohydrates and various glycoconjugates. In plants, GTs are involved in numerous essential processes, including cell wall biosynthesis and post-translational modifications. GTs are classified into over 130 distinct gene families [1]. The basic structure of type II transmembrane GTs includes a short N-terminal cytosolic tail, a transmembrane domain (TMD), a flexible stem region, and a large catalytic domain [2,3]. A second group of GTs includes enzymes with multiple TMDs and a large catalytic domain, typically positioned on the cytosolic side of the membrane [3,4]. These enzymes often have an additional function, translocating elongating polysaccharides across the membrane, either into the Golgi lumen or the apoplastic space over the plasma membrane. These structural arrangements enable GTs to maintain proper orientation within cellular membranes while performing their catalytic functions in glycosylation processes.

The plant cell wall is composed of structurally diverse polysaccharides and glycoproteins, forming a complex network that serves as a barrier to protect plants and cells against environmental cues [5]. These dynamic structures play crucial roles in supporting the cell’s structure, controlling cell shape, regulating growth, and facilitating communication with the environment [5,6]. The main polysaccharides forming the primary structural elements in most cell walls are cellulose, hemicelluloses, and pectin [5,6,7,8]. GTs involved in the biosynthesis of cell wall polysaccharides are localized in the Golgi and plasma membrane [3,7,8,9,10,11,12,13]. During this process, GTs exhibit diverse organizational structures, often forming functional homodimers, heterodimers, or multiprotein complexes through protein-protein interactions [3]. These various protein dimers and larger complexes are essential for the coordinated synthesis of the complex and highly branched cell wall polysaccharides. Protein-protein interactions are also critical for regulating enzymatic activity and achieving the precise spatial organization required for assembling intricate cell wall structures with diverse branching patterns.

O-glycosylation and N-glycosylation are fundamental post-translational modifications and the primary types of glycosylation involving GTs. O-glycosylation of proteins occurs most frequently at serine and threonine residues, primarily in the Golgi, but also in the cytoplasm and nucleus [14,15,16,17]. In plants, sugars are primarily attached to the oxygen atoms of hydroxyproline (Hyp) residues in proteins, including arabinogalactan proteins (AGPs), extensins (EXTs), and repetitive Pro-rich proteins (PRPs) [18]. Galactosyltransferases are involved in the elongation of the side-chain backbone of AGPs, while arabinosyltransferases add arabinose residues to Hyp in Hyp-rich glycoproteins (HRGPs) [17,18]. In serine and threonine O-glycosylation, SPINDLY (SPY) (O-fucosyltransferase) adds O-fucose, and SECRET AGENT (SEC) (O-GlcNAc transferase) adds O-linked N-acetylglucosamine to serine or threonine residues [19,20,21]. Other GTs, such as glucosyltransferases from the UGT72 family, glycosyltransferases from the UGT84 family, and flavonoid 7-O-glycosyltransferase (CsUGT75L12), are also reported to be involved in O-glycosylation [22,23,24]. Currently, there is no evidence that GTs involved in O-glycosylation are assembled into multiprotein complexes, and no interactions between these proteins have been reported so far.

In the N-glycosylation process, the first sugar of the glycan is attached to specific asparagine residues of proteins. This process is essential for cell viability, protein solubility, structure, and function. N-glycosylation occurs at asparagine residues within the consensus sequence Asn-X-Ser/Thr (where X can be any amino acid except proline) [18,25,26]. The biosynthetic process of N-glycosylation has two main phases: the Initial assembly of oligosaccharides and their transfer in the Endoplasmic Reticulum (ER) [27], followed by further elongation and structural diversification of complex glycans through processing in the Golgi apparatus [18,25,26]. In the ER, the precursor oligosaccharide is transferred to the peptide’s Asn by the oligosaccharyltransferase (OST) complex [26,27]. α-glucosidases I and II (GSC1&2) and α-mannosidase 3 (MNS3) initiate trimming in the ER [28,29]. In the Golgi, ER-mannosidase (MNS3) and GMI continue mannose trimming [28,30]. The *cis*/*medial*-Golgi N-acetylglucosaminyltransferase I (GNTI) adds N-acetylglucosamine (GlcNAc) to Man5GlcNAc2 N-glycan [31]. Plant-specific glycosyltransferases, including β1,2-xylosyltransferase (XYLT) and core α1,3-fucosyltransferase (FUT), are also involved in further modifications [32,33,34,35]. β1,3-galactosyltransferase (GALT1) and α1,4-fucosyltransferase (FUT13) are specifically involved in synthesizing Lewis A-type structures on N-glycans [26,36,37].

In this review, we discuss the protein-protein interactions, complex assembly, and dimerization of GTs involved in hemicelluloses, pectin biosynthesis, and N-glycosylation. Given the recent publication of several comprehensive reviews on GTs involved in cellulose biosynthesis [8,38,39], we have chosen not to reiterate this information. By elucidating the mechanisms of protein-protein interactions between GTs and the assembly of their complexes, our goal is to provide the newest insights into the sophisticated glycosylation machinery responsible for synthesizing complex polysaccharide networks and post-translational glycosylation.

## 2. Protein-Protein Interactions Among GTs Involved in Polysaccharide Synthesis and Protein Glycosylation in Golgi

### 2.1. Xyloglucan Synthase Complex

**Figure 1:**
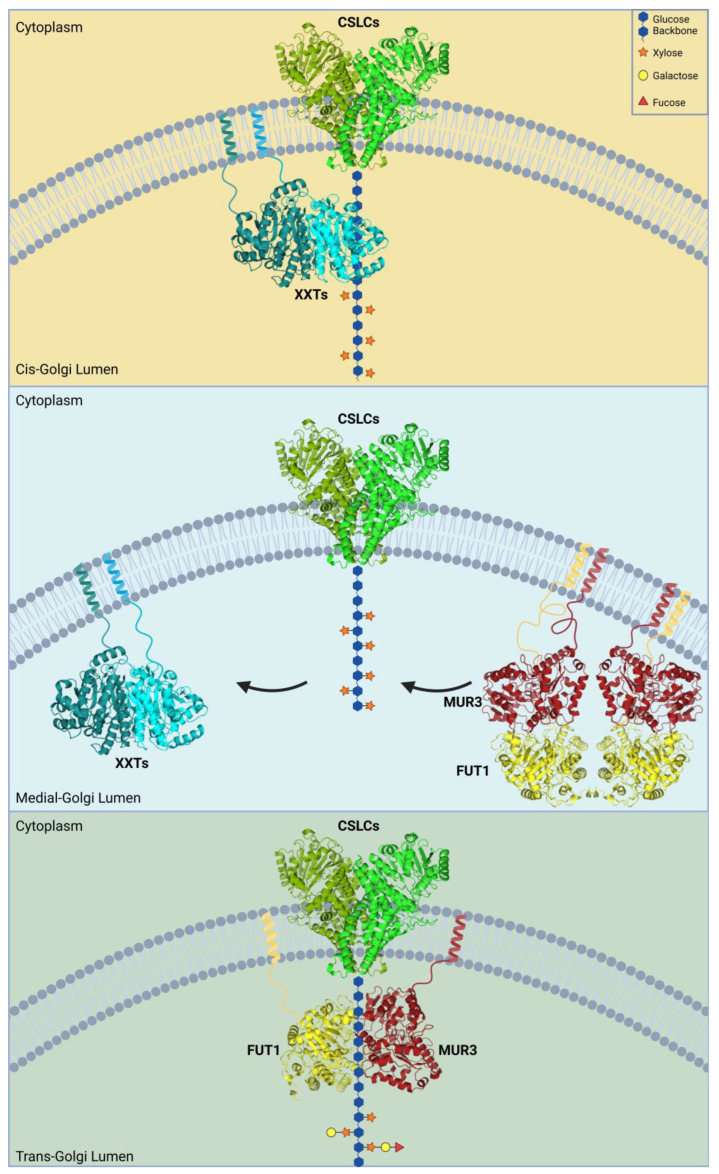
Model of transient formation of XyG-synthesizing complex. CSLCs likely act as anchors for polysaccharides and recruit other enzymes. XXTs likely bind to the glucan backbone in the *cis*-Golgi and are released in the *medial*-Golgi. MUR3 and FUT1 then likely replace the XXTs in the *medial*-Golgi, fully synthesizing the XyG polysaccharide through the *trans*-Golgi. Once completed, the polysaccharide is transferred from the *trans*-Golgi to the apoplast via vesicles. Crystal structures of XXT1 (PDB ID: 6BSU) and FUT1 (PDB ID: 5KOP) were used alongside in silico-generated structures of CSLC4 and MUR3. CSLCs include CSLC4, CSLC5, CSLC6, CSLC8, and CSLC12, while XXTs include XXT1, XXT2, and XXT5. The homodimerization of CSLCs and the hetero-oligomerization of MUR3-FUT1 were generated using AlphaFold Multimer. The figure is created in Biorender.com.

To date, the most well-understood glycosyltransferases (GTs) in terms of protein-protein interactions and dimeric structure formation are the xyloglucan (XyG)-synthesizing GTs. XyGs are common hemicelluloses found in most plants, but they are more abundant within eudicot cell walls. All XyGs are composed of a β-1,4-glucan backbone that is α-1,2-xylosylated (Figure 1) [10,40]. These xylosyl residues are often further expanded with a wide variety of sugars, including galactose, arabinose, fucose, and glucuronic acid, depending on the plant species or specific tissues [41,42,43,44,45]. XyGs exhibit high biodiversity in their glycan composition among different plant species, carrying unique monosaccharides and patterns within their structures [42,43,44,45]. A single-letter nomenclature has been introduced and is continuously updated to reflect variations in substitutions found in numerous plant XyGs [46,47]. Unbranched glucose residues are denoted by G, while xylosylated glucan is denoted by X. Typically, XyG is broken down into a mixture of repetitive subunits, determined by the number of xyloses substituting the glucan backbone. In *Arabidopsis thaliana* (*A. thaliana*), for instance, these subunits share core features of a tri-xylosylated glucan backbone followed by a single unbranched glucose [10,12]. This repetitive subunit, referred to as XXXG, can be further branched by galactose and fucose in vegetative tissue [10,12]. The second and third xylosyl residues are β-1,2-galactosylated, denoted by L, forming XLLG. Finally, an α-1,2-fucose binds to the third branch of the subunit to form the most complex XLFG subunit [10,12]. in *A. thaliana* root tissues, there is a unique form of branching involving galacturonic acid on the first and third xylose residues [48].

Most studies on protein structures and protein-protein interactions have been conducted using *A. thaliana* XyG-synthesizing GTs. Fourteen GTs have been predicted to be involved in the synthesis of XyG, although many serve as redundant isoforms likely exhibiting tissue-specific roles. Five glucan synthases (CSLCs)—CSLC4, CSLC5, CSLC6, CSLC8, and CSLC12—synthesize the β-1,4 glucan backbone, but protein interaction studies have primarily focused on CSLC4 (Figure 1) [49,50,51]. XyG xylosyltransferases (XXTs) 1 and 2 transfer xylose to the first and second positions, while XXTs 3, 4, and 5 xylosylate the third position [52,53,54,55,56]. XLT2 and MUR3 galactosylate the second and third xyloses specifically to form XLXG and XXLG, respectively [57,58]. Finally, FUT1, the only known XyG fucosyltransferase, fucosylates the galactose in the third branch of the XyG subunit to form XLFG. At least seven diverse enzymes are required to synthesize fully branched XyGs: a single CSLC, two types of XXTs, XLT2, MUR3, and FUT1. CSLCs are predicted to be integral membrane proteins, while the other GTs are all type II membrane proteins.

It is proposed that these seven enzymes likely form a higher-order complex to efficiently synthesize the highly branched structure of XyGs in the Golgi. Protein-protein interactions have been demonstrated between almost all GTs involved in the synthesis. CSLCs are believed to form the core of the oligomeric structure, synthesizing the nascent glucan backbone and recruiting the other GTs. Live-cell imaging studies showed that CSLC4 forms a homodimer and interacts with all XyG-synthesizing GTs except XXT1 [50,59,60] (Table 1). additional interactions between several GTs in the XyG biosynthetic pathway were observed, including an XXT2-XXT5 dimer, MUR3-FUT1 dimer, FUT1-XXT2 dimer, and FUT1-XXT5 dimer (Table 1). Later, XXT3 was also shown to interact with XXT2 [56]. Further investigations using pull-down assays and Co-immunoprecipitation (Co-IP) of truncated and full-length proteins confirmed most of these interactions [50,59].

Two hypotheses can be proposed for XyG-synthesizing complex organization: (1) formation of a single large complex that includes all enzymes required to build a fully branched XyG molecule, and (2) transient formation of small complexes with diverse compositions. The first model suggests that all enzymes form a large oligomeric complex in the *cis/medial*-Golgi to synthesize a large XyG polymer, remaining together until the fully synthesized polysaccharide is released and transported from the *trans*-Golgi into vesicles. The transient complex model suggests that only a few GTs form smaller oligomeric complexes at a time to synthesize the nascent polysaccharide as it moves through the Golgi cisternae. New GTs are then recruited and likely replace the enzymes involved in earlier steps of synthesis (Figure 1). To date, the latter hypothesis is supported by a larger body of evidence. A study using *Nicotiana tabacum* plants expressing fluorescently labeled XyG-synthesizing proteins XXT1, MUR3, and FUT1 showed distinct distributions of these enzymes across separate Golgi cisternae. XXT1 was detected mostly in the early *cis*- and *medial*-Golgi, while MUR3 and FUT1 predominated in the *medial*- and *trans*-Golgi [61]. Another study using TEM and electrophoretically separated Golgi cisternae indirectly confirmed such differential distribution of GTs. antibodies specific to XyG epitopes detected xylosylated, galactosylated, or fucosylated side chains. Unbranched XyG was primarily found in the *cis*- and *medial*-Golgi, while larger branched XyG was detected in the *medial*- and *trans*-Golgi [62]. These results suggest a shift from predominantly XXTs-containing protein complexes to MUR3-FUT1 enriched complexes within the *medial*-Golgi cisternae.

**Table 1:** Summary of the hetero-/homo dimers of GTs.

Xyloglucan Synthase Complex			
Protein	Heterodimer With	Homodimer	
AtCSLC4	AtXXT2, AtXXT5, AtXLT2, AtMUR3, AtFUT1	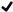	[50,59,60]
AtXXT1	AtXXT2, AtXXT5	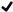	[50,59,60,63]
AtXXT2	AtCSLC4, AtXXT1, AtXXT5, AtXLT2, AtFUT1	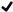	[50,59,60]
AtXXT5	AtCSLC4, AtXXT1, AtXXT2, AtXLT2, AtFUT1		[50,59,60]
AtXLT2	AtCSLC4, AtXXT2, AtXXT5, AtFUT1		[59,60]
AtMUR3	AtCSLC4, AtFUT1		[59,60]
AtFUT1	AtCSLC4, AtXXT2, AtXXT5, AtXLT2, AtMUR3	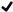	[59,60,64,65]
Xylan Synthase Complex			
Protein	Heterodimer With	Homodimer	
AoIRX9	AoIRX14	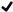	[66]
AoIRX10		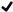	[66]
AoIRX14	AoIRX9	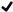	[66]
TaGT43-4	TaGT47-13, TaGLP, TaVER2, TaGT75-3, TaGT75-4	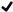	[67]
TaGLP	TaGT43-4	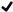	[67]
TaGT75-3	TaGT43-4	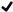	[67]
TaGT75-4	TaGT43-4	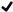	[67]
OsGT43B	OsGT47-2		[68]
OsGT43F	OsGT47-4	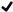	[68]
OsGT43I	OsGT47-1, OsGT47-3	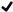	[68]
OsGT43J	OsGT47-2	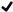	[68]
Pectin Synthase Complex			
Protein	Heterodimer With	Homodimer	
AtGAUT1	AtGAUT5, AtGAUT6, AtGAUT7	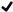	[69,70,71]
AtGAUT10, ATGAUT13, AtGAUT14		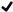	[70]
Arabinogalactan synthesizing GTs			
Protein	Heterodimer With	Homodimer	
AtARAD1	AtARAD1	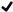	[72]
AtARAD2	AtARAD2	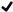	[72]
NaARAD1	AtARAD1	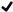	[73]
AtGALT29A	AtGALT14A, AtGALT31A	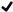	[74]
AtGALT31A		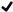	[74]
Proteins involved in N-glycosylation			
Protein	Heterodimer With	Homodimer	
GNTI	MNSI	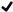	[75,76]
XYLT	GMII	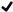	[75,76]
GALTI	GMII		[75]

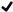
 check mark indicates the confirmation of protein homodimer.

### 2.2. Xylan Synthase Complex

Xylan is a major hemicellulose component in the cell wall of monocots and most woody eudicot plants. The biosynthesis of the β-1,4-linked xylose backbone, which forms the core structure of Xylan, is catalyzed by proteins in the GT43 and GT47 protein families. In rice, wheat, and asparagus, interactions between members of the GT43 and GT47 protein families have been confirmed in vivo and in vitro [66,67,68,77]. These interactions appear to be protein-specific, with OsGT43I specifically interacting with OsGT47-1 and OsGT47-3 [68] (Table 1). While not all members of the GT43 family form homodimers, homodimerization is a common feature observed among many GT43 proteins in the ER [66,67,68] (Table 1). Another example of GTs from the GT47 protein family is AoIRX10, which was shown to form homodimers in asparagus [66] (Table 1). However, similar studies on GTs from the same GT47 family in rice and wheat failed to detect their homodimerization [67,77] (Table 1). Additionally, a Bimolecular fluorescence complementation (BiFC) assay using wheat proteins showed heterodimer formation between TaGT43 proteins and non-GT proteins. For instance, TaGT43-4 interacts with germin-like protein TaGLP, forming a heterodimer with vernalization-related protein TaVER2, and also exhibits strong protein-protein interactions with mutases such as TaGT75 [67,77] (Table 1).

**Figure 2:**
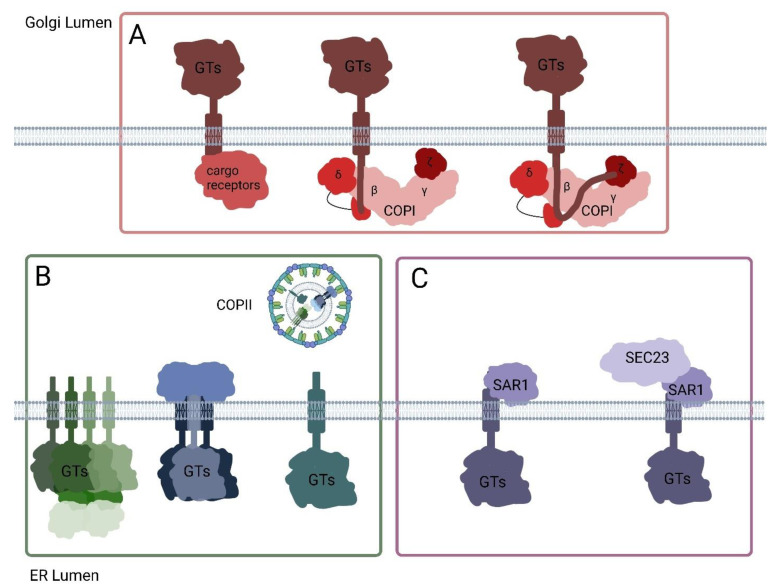
The mechanism of GTs and GTs protein complex trafficking between the ER and Golgi. (**A**): The cargo sorting signal motifs in the cytosolic tail of GTs interact with cargo receptors such as Vps74p and GOLPH3. These signal motifs also interact with the MHD domain of δ-COP, β-COP, and/or ζ-COP. (**B**): GTs and protein complexes (GTs and other assembled proteins) in the ER are transported as a whole via COPII vesicles. (**C**): Protein–protein interactions occur between GTs and COPII coatomers, such as SAR1 and/or SEC23. The figure was created in Biorender.com.

**Figure 3:**
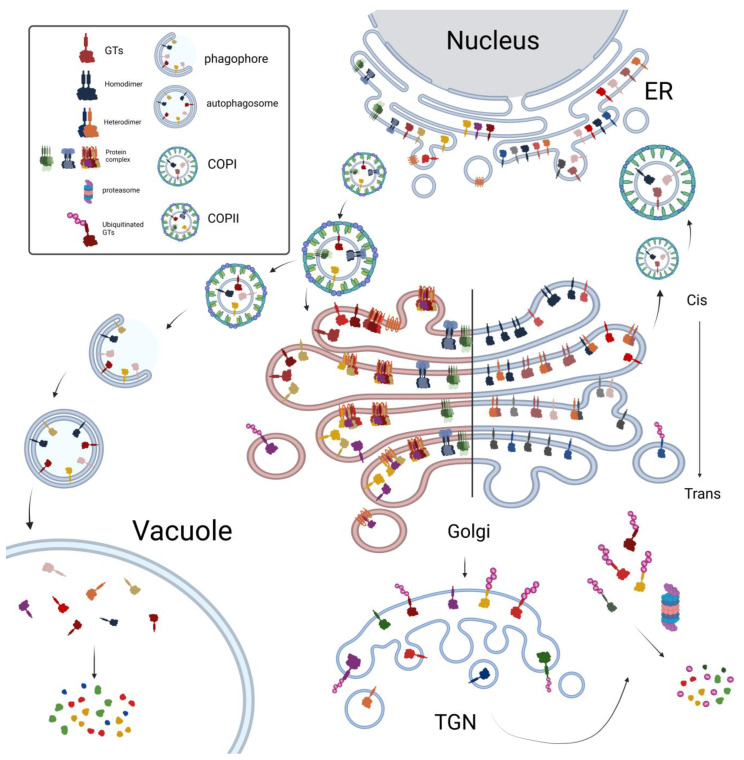
Model for the assembly and trafficking of the GTs and protein complex. Some individual monomer GTs are packed into COPII vesicles through interactions with COPII component proteins and transported to the Golgi. Additionally, certain GTs assemble into protein complexes, which are also packed into COPII vesicles and transported to the Golgi. The right (blue) side of the model illustrates the distribution of GTs involved in N-glycosylation, while the left (red) side depicts the distribution of GTs involved in polysaccharide biosynthesis. Two well-known plant protein degradation pathways are also shown. In the ubiquitination pathway, the ubiquitination of GT proteins can lead to their sorting from the TGN and subsequent degradation by the proteasome. In the Autophagy pathway, GTs can be transported via COPII vesicles to the autophagosome and subsequently delivered to the vacuole for protein degradation. The figure was created in Biorender.com.

The GT43 and GT47 proteins, such as TaGT43-4 and TaGT47-13, form the Xylan Synthase core complex. It has been proposed that these interactions trigger the export of a protein complex from the ER to the Golgi (Figure 2B), contributing to the formation of putative Xylan synthase complexes [66,67,68]. The expression of AoIRX9 alone, and the co-expression of AoIRX9 with AoIRX10 or AoIRX14A, resulted in partial Golgi and ER localization. However, the co-expression of all three proteins (AoIRX9, AoIRX10, and AoIRX14A) led to a predominantly Golgi-localized protein complex [66] (Figure 2B). This finding indicates that the assembly of the AoIRX9, AoIRX10, and AoIRX14A protein complex is a prerequisite for accurate Golgi localization (Figure 3). Homologous to AtIRX14, the TaGT43-4 protein serves as a scaffold to interact with TaGLP, TaVER2, and TaGT75 proteins in the ER, assembling the Xylan synthase complex [67] (Figure 2B and Figure 3). Furthermore, the interaction between TaGT43-4 and TaGT47-13 facilitates the export of the complex from the ER to the Golgi [67].

### 2.3. Pectin Synthase Complex

Pectin is a complex heteropolysaccharide that plays a crucial role in plant cell walls and has significant importance in regulating plant growth and stress responses. These heteropolymers also have extensive utilization in various industrial applications. As one of the major components of the primary cell wall in plants, pectin accounts for up to 35% of the cell wall composition in eudicots and non-graminaceous monocots [11]. The GTs involved in pectin biosynthesis belong to glycosyltransferase family 8 (GT8) and are responsible for catalyzing the transfer of galacturonic acid (GalA) residues from UDP-GalA to growing pectin chains [78]. *A. thaliana* galacturonosyltransferase (GAUT) 1 and its homologous GAUT7 protein are α-1,4-galacturonosyltransferases that synthesize homogalacturonan (HG). Both GAUT1 and GAUT7 are type II membrane proteins with N-terminal, TMD, stem region, and catalytic domain [69]. However, the GAUT1 protein undergoes post-translational cleavage of its N-terminal and TMD (Met1 to Ala169) and therefore requires GAUT7 to be retained in the Golgi [69]. It was confirmed that GAUT7 recruits the cleaved GAUT1 from the ER to the Golgi via direct protein-protein interaction [69,70,71,79]. In addition, GAUT5 and GAUT6 were demonstrated to exhibit a function similar to GAUT7 in anchoring GAUT1 [70,71] (Table 1). GAUT1 forms a homodimer to interact with GAUT7, assembling the pectin synthase core complex [69,70,71,79]. Co-expression of GAUT proteins 3–15 with GAUT1 in HEK293F cells demonstrated in vitro evidence that GAUT1 likely interacts with GAUT5, GAUT6, and GAUT7 [70]. Utilizing GFP-fused proteins, GFP-GAUT1 emitted higher fluorescence and, therefore, higher expression when co-expressed with non-fused GAUT7 compared to GFP-GAUT1 expression alone. The same was observed for GAUT5, GAUT6, and GAUT7 when either GFP-fused protein was co-expressed with non-fused GAUT1. Non-reducing SDS-PAGE analysis also corroborated the formation of the GAUT1-GAUT7 heterocomplex as well as homo-complexes of GAUTs 10, 13, and 14 [70]. Additionally, GAUT1 and GAUT7 work cooperatively in vitro to produce high-MW polysaccharides when sufficient GalA is present [79]. Based on these results, a hypothetical model of distributive HG elongation was proposed, suggesting that GAUT7, together with GAUT1, is required for HG biosynthesis. GAUT7 may assist in forming the extended acceptor-binding groove and strengthen the charged interactions between longer-chain acceptors and the GAUT1:GAUT7 complex [79]. These results demonstrate that heterocomplexes are preferred by some GAUT GTs, likely improving the stability and efficiency of the enzymes.

### 2.4. Arabinogalactan Synthesizing GTs

Arabinogalactan is a complex biopolymer composed of arabinose and galactose monosaccharides in plants. There are three main types of arabinogalactan structures: type I arabinogalactan, type II arabinogalactan, and arabinogalactan associated with pectic polysaccharides. In pectin biosynthesis in *A. thaliana*, the arabinan arabinosyl transferase (ARAD1) and its homolog ARAD2 form heterodimers, possibly through disulfide bridges [72]. In microsomes prepared from *Nicotiana benthamiana* (*N. benthamiana*) transiently expressing ARAD1-Myc-6xHis and ARAD2-YFP, heterodimers of ARAD1 and ARAD2, as well as homodimers of ARAD1 and ARAD2, were detected by immunoblotting [72] (Table 1). In *Nicotiana alata*, the NaARADL1 protein also forms homo- and heterodimers with AtARAD1 [73]. The GT29 family member AtGALT29A and other arabinogalactan GTs, including AtGALT31A and AtGLCAT14A, are involved in the biosynthesis of type II arabinogalactan [74]. Co-localization studies showed that 80% of AtGALT29A co-localized with AtGALT31A, while 52% co-localized with AtGLCAT14A [74]. AtGALT29A expressed in *N. benthamiana* interacted with AtGALT31A in the Golgi, and protein-protein interactions between AtGALT29A and AtGALT31A were confirmed using Co-immunoprecipitation (Co-IP) and Förster resonance energy transfer (FRET) assays [74] (Table 1). AtGALT29A was reported to occasionally interact with AtGLCAT14A, though with a lower frequency compared to its interaction with AtGALT31A [74] (Table 1). The presence of heterodimers of AtGALT29A and AtGALT31A contributed to an increased level of galactose incorporation during type II arabinogalactan biosynthesis. The interaction between AtGALT29A and AtGALT31A boosted the enzymatic activity of β-1,6-galactosyltransferase in adding galactose residues at O6 positions of β-1,6-galactan and β-1,3-galactan [74]. AtGALT29A and AtGALT31A can also form homodimers, and their protein-protein interactions were detected by FRET [74].

### 2.5. Proteins Involved in N-glycosylation

N-glycosylation is a fundamental and widespread post-translational modification that occurs in plant cells. N-glycosylation involves the attachment of glycans to specific asparagine residues within proteins. N-glycosylation plays crucial roles in protein folding, protein stability, protein trafficking, and molecular recognition. Plant N-glycosylation differs from animal N-glycosylation in several key ways [18]: plant N-glycans completely lack sialic acid; core fucose residues in plant N-glycans are α (1→3)-linked to the reducing GlcNAc, whereas in animals, they are α (1→6)-linked. Additionally, plant N-glycans often have a β (1→2)-linked xylose attached to the core β-mannosyl residue, which is not present in animal N-glycans. The N-glycosylation process occurs in two main phases: initial synthesis in the ER and subsequent modifications of the glycan chain in the Golgi apparatus. In the early stage of N-glycosylation in the Golgi, the *cis/medial*-Golgi-localized alpha-mannosidase I (MNS1) and β1,2-N-acetylglucosaminyltransferase I (GNTI) interact, forming a heterodimer, as shown in both in vivo and in vitro studies [75]. The *medial*-Golgi β1,2-xylosyltransferase (XYLT) and alpha-mannosidase II (GMII) assemble into heterodimers, and the *trans*-Golgi-localized β1,3-galactosyltransferase1 (GALT1) strongly interacts with GMII [75] (Table 1). In addition, broad homodimerization among GTs involved in N-glycosylation has also been observed. For example, GNTI [75,76] and XYLT [76] are able to form homodimers. In both homodimerization and heterodimerization (Table 1), the cytoplasmic-transmembrane-stem region (CTS) of these GTs mainly contributes to the protein-protein interactions [75,76]. For example, it was shown that the CTS region of GNTI interacts with the CTS region of both GNTI and GMII. Similarly, interactions in the XYLT homodimer and the XYLT-GMII heterodimer occur through their CTS domains [75]. The most recent studies revealed that the specific three amino acids “ALE” in the stem region of GNTI contribute to the homodimerization of this protein [76]. deletion of these three amino acids (“ALE”) in the stem region of GNTI mostly abolished the protein-protein interactions of the mutant GNTI, which failed to form a homodimer [76].

GTs can be distributed in different Golgi cisternae to process more complex branching or elongation of glycans. Such distribution patterns have been observed for GTs involved in protein N-glycosylation and XyG biosynthesis (Figure 3). *Cis*-Golgi alpha-mannosidases MNS1 and MNS2 localize in the early stacks of the Golgi and remove Man residues from Man8GlcNAc2 to produce Man5GlcNAc2. Next, the *cis*-Golgi GNTI transfers a GlcNAc residue to the Man5GlcNAc2 substrate to synthesize GlcNAcMan5GlcNAc2 glucan [31]. The products generated by GNTI undergo two potential modifications: further trimming by *medial*-Golgi mannosidase II (GMII) or serving as the acceptor substrate for *medial/trans*-Golgi XYLT [18,26,75]. Subsequently, GNTII and FUT11/12 continue further modification of the N-glycan. To synthesize Lewis A-containing structures, *trans*-Golgi GALT1 catalyzes the addition of β1,3-linked galactose residues to N-glycan acceptor substrates, and FUT13 adds an α1,4-linked fucose residue to the galactose added by GALT1.

### 2.6. Structural Analysis of the Homo- and Heterodimerization of GTs

To date, few structures of plant GTs involved in the glycosylation processes described in previous sections have been solved. No protein structures are available for the GTs involved in N-glycosylation in plants. Enzymes in this biosynthetic process have been studied in other eukaryotes, such as yeast and mammals, and their 3D structures have been covered extensively by other reviews. In cell wall polysaccharide biosynthesis, four enzymes have been structurally characterized at the time of writing this review: FUT1, XXT1, GalS1, and CSLF6 [63,64,80,81,82]. The crystal structure of XyG-biosynthesizing FUT1 depicted a homotetramer, although fast protein liquid chromatography (FPLC) analyses suggest that FUT1 is a heterodimer in vivo [64,80].

Similarly, XXT1 was also found to be a homodimer through both crystallization and FPLC analyses [63]. The XXT1, XXT2, and XXT5 proteins share high sequence similarity, suggesting that these GTs are likely to have a similar overall fold. Indeed, homology models and publicly available AlphaFold predictions (https://alphafold.com/) [83] for XXT2 and XXT5, constructed using the crystal structure of XXT1, demonstrated very close folding [63]. Therefore, it is plausible that XXT2 and XXT5 interact together, forming a heterodimer in a manner similar to the homodimer characterized for XXT1. This notion requires experimental confirmation.

In rhamnogalacturonan I biosynthesis, GalS1, the only GT to be structurally characterized, is involved in the elongation of rhamnogalacturonan I side chains and capping by galactose and arabinose, respectively. GalS1 was crystallized as a homotetramer but also exists as a dimer in vitro [82].

The integral membrane protein CSLF6 has a different topology with six TMDs and is involved in the synthesis of β-1,3;1,4-mixed glucans. This glucan synthase is the first structure from the cellulose synthase-like family of proteins, which are predicted to be closely related to cellulose synthases CesA and CesB and belong to the same GT2 protein family. Uniquely, this enzyme functions as a monomer, and when mixed with differently tagged CSLF6 enzymes, they did not interact to form a higher-order structure. This observation suggests that, in contrast to the CSLCs and CESA proteins, this glucan synthase may not be involved in protein-protein interactions [81].

## 3. The Trafficking of GTs Between ER and Golgi and Their Interaction with Coat Proteins

The trafficking of GTs through the endomembrane system of cellular compartments is a complex process that follows established secretory pathways similar to other membrane proteins [84]. This trafficking is primarily mediated by two major coat protein complexes: COPI and COPII, which facilitate transport between the ER and Golgi apparatus. The COPI complex consists of seven subunits (α, β, β’, γ, δ, ε, and ζ-COP) and requires activation of GTPase Arf1 for coat assembly, while COPII-mediated transport involves Sar1 GTPase activation and assembly of the coat protein complexes Sec23-Sec24 and Sec13-Sec31. The cargo sorting process is critical during the assembly of COPI and COPII complexes and heavily depends on specific sorting signals present in cargo proteins. These signals are fundamental in determining whether cargo proteins interact directly or indirectly with coat components and further ensure accurate protein trafficking, the maintenance of proper protein organization, and their cellular localization.

Studies in *N. tabacum* have revealed the importance of specific amino acid residues in the cytosolic tail of Golgi-localized N-acetylglucosaminyltransferase I (GNTI). The cytosolic tail sequence MRGYKFCCDFR contains key arginine residues that affect protein localization [85]. When R11 and K5 were mutated, GNTI was found in both the ER and Golgi. A Triple mutation of R11, K5, and R2 caused GNTI to predominantly remain in the ER, while mutation of only K5 and R2 resulted in exclusive Golgi localization. These findings indicate that R11, positioned close to the transmembrane domain, is crucial for Golgi retention of GNTI [85]. The highly conserved glutamine (Q) residue within the TMD sequence of GNTI in *N. tabacum* also participates in protein trafficking and localization. Mutation of Q25 to alanine (A) in the conserved sequence FIYIQ in the TMD caused mislocalization of NtGNTI from the *cis/medial*-Golgi to the *trans*-Golgi [85]. Similar amino acid requirements were observed in *A. thaliana*: lysine and arginine residues in the cytosolic tails of GMII and XYLT determine the subcellular Golgi localization of these enzymes [85]. The TMD of AtGNTI also plays a critical function in Golgi localization. The mutation of Q23 to A in the TMD of AtGNTI resulted in the mislocalization of mutated AtGNTI to the apoplast instead of the Golgi [86]. Mutation of Q23 to A shortened the half-life and reduced the stability of AtGNTI, leading to a loss of function in the synthesis of complex N-glycans in *gntI* mutant plants [86]. Co-expression studies in *N. benthamiana* leaf epidermal cells demonstrated that the N-terminal domain of GNTI and Sar1p co-localize at specific punctate structures known as ER-exit sites (ERES) [85]. However, when the basic amino acids in GNTI’s cytoplasmic tail were mutated, the protein lost its ability to recruit Sar1 to these ERES locations [85] (Figure 2C). This finding provides strong evidence that potential protein-protein interactions between GNTI and COPII vesicle component proteins play a crucial role in the transport of GNTI from the ER to the Golgi apparatus in plant cells.

The specific amino acid residues or sequence motifs are broadly reported to be critical in the trafficking and localization of GTs via direct interaction with cargo receptors and COPI/COPII-coated proteins. While these mechanisms have been extensively studied in yeast and mammalian cells, similar investigations in plants are currently scarce. Therefore, we highlight key findings from yeast and mammalian cell studies. In *Saccharomyces cerevisiae*, numerous GTs share a common consensus sequence: (F/L)-(L/I/V)-X-X-(R/K), which plays a crucial role in GT trafficking by interacting with the cargo receptor Vps74p [87] (Figure 2A). For example, α1,2-mannosyltransferase (Kre2p) contains the FLSKR motif in its cytosolic tail. Single mutations of F4 or L5 and double mutations of K7 and R8 in this FLSKR motif disrupted the protein-protein interaction between Vps74p and Kre2p [87]. In animal cells, GOLPH3, the ortholog of yeast Vps74p, performs a similar function in controlling GT localization and trafficking. GOLPH3, as a cargo receptor, directly interacts with β1,6-N-acetylglucosaminyltransferase-1 (C2GNT1) and α-2,6-sialyltransferase 1 (SiaTI) in vitro, and GOLPH3, C2GnT1, and SiaTI have been detected in COPI vesicles [88] (Figure 2A). GOLPH3 interacts with cargo proteins via both the LxxR motif and positively charged amino acids upstream of this motif [89]. This interaction retains the cargo proteins in the Golgi cisternae and prevents their premature exit to the trans-Golgi network (TGN) [89,90]. The Golgi protein GlcNAc-1-phosphotransferase (Ptase) was confirmed to interact with δ- and ζ-COP subunit proteins by BioID2 and pull-down assays [77]. Ptase directly binds to the highly conserved sequence VRFSTE in the MHD domain of δ-COP (Figure 2A). The interaction between Ptase and δ-/ζ-COP is weakened by mutations of K4 to Q, R8 to G, and S15 to Y in the cytosolic tail of Ptase [91]. Additionally, C2GNT1 and N-acetylgalactosaminyltransferase (GALNT) proteins interact with β-COP, ζ-COP, and the MHD domain of δ-COP via the φ-(K/R)-X-L-X-(K/R) sequence in their cytosolic tails [91] (Figure 2A). The WTW motif in the cytosolic tail of GALNT4 is responsible for its interaction with the MHD domains of δ-COP and β-COP, despite the absence of a φ-(K/R)-X-L-X-(K/R) sequence in GALNT4’s cytosolic tail [91]. In addition, the Sar1 and Sec23p proteins, which are involved in the assembly of COPII vesicles, have been proven to interact directly with GTs. Synthetic cytosolic tails containing RR motifs from Polypeptide N-acetylgalactosaminyltransferase (GalNAcT) and Hydroxyproline-O-galactosyltransferase 2 (GALT2) demonstrated interaction with Sar1 and Sec23p in vitro [92] (Figure 2C). mutation of RR to AA impaired the interaction between GTs and Sar1, and the presence of active Sar1 increased protein interactions between GTs and Sec23p [92].

## 4. Future Directions

In our review, we have covered only a small percentage of known GTs within plants. GTs and their functions, especially those in plants, are vastly understudied in comparison to other plant biosynthetic processes. The function and organization of numerous GTs in plants are still unknown, and many of those that have been identified or predicted still lack biochemical confirmation of their specific activity or structural organization. broad and intensive studies using recombinant proteins and their co-expression are needed to fully understand the structural organization, functions, and importance of multiprotein complexes of GTs. Although some recent strides have been made in both protein-protein interaction and structural characterization, the structures of plant GT heterocomplexes have yet to be reported. In other fields, protein structural prediction software such as AlphaFold has become an effective tool for the quick, accurate estimation of structure and substrate or inhibitor binding. Even more significantly, AlphaFold Multimer has been developed to predict how multiple proteins may interact with one another, regardless of whether they actually interact or not. Paired with a multitude of high-throughput techniques, protein characterization is moving at a much faster pace. For glycobiology, specifically in plants that synthesize some of the most complex and diverse polysaccharides in nature, in silico tools will likely prove to be a significant advantage in the future. However, caution must be taken in the broad use of these bioinformatic tools in understanding GTs and their complexes. These tools use empirical data from other GTs to calculate their predictions. At the time of writing, there is still a big gap in the functional characterization of many GTs, and the lack of structural data may mislead users. To reiterate, many CaZY families lack biochemical and structural characterization, and only four plant cell wall GTs have been reported. A significantly higher variety of plant GT structures is needed to not only improve our understanding of plant GTs and polysaccharide synthesis but also refine and develop modern software for more accurate predictions.

To further complicate matters, demonstrations of the promiscuity of GTs in in vitro assays often conflict with in vivo observations from reverse genetics and analysis of cell wall extracts. For example, it has been demonstrated that AtXXT1 can bind and catalyze the transfer of sugars other than xylose [93]. AtXXT1 has also been reported to synthesize tri- and tetra-xylosylated glucan via in vitro assays [94]. However, neither observation has been reported by any in vivo studies. In vivo studies report that XXT1 can only xylosylate two glucoses in a row (XXGG), and no substitutions of xylose have been detected [56]. Similar observations are plausible for recombinant heterocomplexes and illustrate the value of in vivo confirmation. Efforts in this area of plant research would support significant advances in plant GT studies and provide insight into how plants synthesize complex and diverse polysaccharide structures that support the critical aspects of plant cell walls.
